# Scarlatina without Fever

**Published:** 1891-01-10

**Authors:** 


### SCARLATINA WITHOUT FEVER.

Dr. Beetz and Dr. Wertheimer, in the *Munch. Med. Wochenschr.*, after referring to cases of scarlatina of so ephemeral a character that their true nature is unrecognised or discovered only by the sequelæ, and others in which the initial fever subsides after a day or two, describe some of much rarer occurrence, that have lately come under their notice, in which the eruption was well marked and the disease ran its regular course, in one being followed by albuminuria, but in which the temperature remained normal throughout. Dr. Wertheimer however, points to one symptom which in his opinion will always serve to distinguish these cases of non-febrile scarlatina from scarlatiniform erythemas, in the fact that the pulse is no less accelerated in frequency than in cases attended by the usual amount of pyrexia. We may here observe that we once met with an erythema absolutely identical with that of scarlatina, but unattended by fever or acceleration of the pulse, in an infant of four months at the breast, the mother having had crabs for supper on two successive nights. On the other hand, the two most striking instances of insidious scarlatina that have come under our notice, in different families, were girls who were believed to have escaped although the disease had been in the house. One aged 12, we first saw in violent uræmic convulsions, from which, however, she recovered; the other died suddenly after carrying her sister upstairs to bed, and a drachm of urine drawn from her bladder was almost solidified by nitric-acid and boiling. In each there was some œdema, but no other symptoms to arouse the suspicions of the parents at the time.

